# Oral Hygiene Practices among Saudi Arabian Children and Its Relation to Their Dental Caries Status

**DOI:** 10.1155/2018/3234970

**Published:** 2018-04-16

**Authors:** M. F. A. Quadri, M. A. Shubayr, A. H. Hattan, S. A. Wafi, A. H. Jafer

**Affiliations:** ^1^Dental Public Health, Jazan University, Jizan, Saudi Arabia; ^2^Department of Preventive Dentistry, Jazan University, Jizan, Saudi Arabia; ^3^Ministry of Health, Riyadh, Saudi Arabia

## Abstract

Dental caries is one of the most common preventable diseases occurring among children. The aim here is to survey the oral hygiene practices that are commonly followed by Arab children and to see its relationship with their dental caries status. A cross-sectional study with multistage random sampling technique was conducted. Sociodemographic data and information on oral hygiene practices like use of toothbrush, dental floss, siwak, frequency of brushing along with number of snack between meals per day, and consumption of sugar per day was obtained. Presence of plaque on tooth surfaces was reported using plaque index, which was followed by DMFT index to determine the dental caries status. Among the sample of 500 school children, the mean plaque score in male (mean = 0.69; SD = 0.50) was slightly higher than the female (mean = 0.66; SD = 0.46). Increased frequency of snacks (*P*=0.05; ß=0.08; CI = −0.00, 0.09) and sugar consumption (*P*=0.01; ß=0.16; CI = 0.04, 0.27) per day significantly showed higher values of DMFT. Also, the odds of dental caries among the school children who were irregular in brushing their teeth was higher in contrast to the children brushing once (*P*=0.03; OR = 0.89; CI = 0.70, 1.12) or twice (*P*=0.03; OR = 0.80; CI = 0.64, 0.93) per day. It is recommended that the dental public health practitioners here should consider the effect of oral hygiene practices on oral health status in order to design the future health promotion interventions.

## 1. Background

Oral health is linked with general health, and diseases occurring in oral cavity are of major public health importance due to their high prevalence and tremendous social impact [1]. Oral diseases in children are reported to affect their everyday activities both at school and at home. Moreover, the psychosocial impact of these diseases often diminishes their quality of life [2]. Dental caries and periodontal diseases are the most frequently diagnosed oral diseases in young individuals [3]. Good oral hygiene practices are recommended by specialists for preventing these oral diseases [4, 5], and studies conducted earlier have reported poor oral hygiene among children to be associated with the onset of dental caries [6–10]. There are also reports which specifically say that brushing twice daily is important in preventing teeth caries [6]. Apart from brushing, the complete concept of oral hygiene like frequency and duration of brushing; time of brushing; flossing; and so on should be studied and improved among individuals of different age groups and also in different parts of the world [11]. For instance, a study conducted in Nigeria portrayed that immediate teeth cleaning after sweet food or beverage consumption decreases the risk of dental caries [12]. Furthermore, it is also reported that fluoride-containing tooth pastes provide a better check on caries when compared to nonfluoridated tooth pastes [11, 13]. Thus, there are many factors that contribute to oral hygiene practices. Likewise, even for initial plaque formation on the teeth of children, factors such as poor diet, improper brushing, and no flossing contributed actively [14].

In Saudi Arabia, the prevalence of dental caries among children ranges from medium to high as stated in the World Oral Health report [14]. Some region-based studies in Saudi Arabia had also indicated high prevalence of dental caries among school going children [15–17]. We assume that there may be variances in the practices of oral hygiene among the population residing in different sectors of the world specially attributed to their culture and beliefs. One example could be the use of siwak (miswak); which is a name given by Arabs alternatively for chewing stick (*Salvadora persica*) [18]. Knowing about oral hygiene practices of children among Arab regions like that of Saudi Arabia will aid in proper design and implementation of oral health promotion programs, thus reducing the prevalence of oral diseases. Gizan, a province of Saudi Arabia is reported with high prevalence of dental caries among school-aged children [16], but there are very less published researches to explain the contributory risk factors. Due to the scarcity of data in this region, proper prevention programs by the department of dental public health at Jazan University and various other governmental authorities are difficult to implement. Hence, the aim here is to survey the oral hygiene practices that are commonly followed by Arab children residing in Gizan region of Saudi Arabia and to see its relationship with their dental caries status.

## 2. Materials and Methods

### 2.1. Ethical Consideration

Permission from the research committee at college of dentistry, Jazan University was obtained. This was followed by the permission from the regional education office to approach the schools. An informed consent was also taken from parents for the participation of their children in the current study.

### 2.2. Study Setting and Population

The study was conducted in Gizan, which is located at the southern tip of Saudi Arabia bordering Yemen. Six different schools and a dental camp organized at a public place were the sites for data collection. Only Saudi Arabian children aged 12 and 13 years were included to maintain the homogeneity of the study population.

### 2.3. Study Design and Sample Size

A cross-sectional study with multistage random sampling technique was conducted in the year 2016. Size of the sample was calculated (*N*=500) with an absolute precision of 0.05, expected proportion of 0.5, and an estimated design effect of 1 [16]. A complete list of schools was obtained from the Ministry of Education, and in the first stage of sampling, out of four districts (Sabya, Jazan city, Abu Arish, and Samtha) in the Gizan region, three were selected (Jazan city, Sabya, and Abu Arish). In the second stage, six schools (three male and three female schools) were randomly selected from the aforementioned areas. The third stage involved simple random selection of high school pupils from both male and female schools using their national identification numbers. To reach the calculated sample size, children attending the annual dental camp were also targeted, and national identification numbers were matched to avoid duplication of the data.

### 2.4. Variables Assessed

#### 2.4.1. Oral Hygiene Practices

Oral hygiene information was collected using a predesigned and prevalidated questionnaire. This self-reported structured questionnaire included the following variables: (i) use of toothbrush, (ii) dental floss, (iii) siwak (Miswak), (iv) frequency of brushing, (v) number of snack between meals per day, (vi) consumption of sugar, and (vii) number of meals per day. The questionnaire was originally formulated in English and then translated into Arabic, and prior to the survey, pretests took place to control reliability and validity (ICC = 0.77) of the questions. Information on demographic details such as age, gender, parent's education, and parent's employment status were self-reported.

#### 2.4.2. Clinical Examination

Clinical examination was conducted by pretrained and precalibrated (interrater = 0.98 and intrarater = 0.98) fours graduating dentists. Dental caries status of the school children was determined using DMFT and plaque indices [1]. The procedure involved careful quadrant by quadrant examination and recording of the findings. One class room in each school was dedicated for examination, where the child was given an ordinary chair to sit, and the examiner embraced the standing position for diagnosis. Each examiner was provided with an assistant to manually record the findings on dedicated questionnaires. Standard infection control practices were maintained, and disposable diagnostic set of instruments were used under the normal light. As recommended by WHO, a decay in tooth was considered if the cavitation stage has been reached, while teeth extracted for reasons other than caries were not considered as missing.

### 2.5. Statistical Analysis

Recorded data were checked for missing values and normality distribution. Obtained values were either summarized as means ± SD or percentages. Correlation between oral hygiene practice and oral health status was tested by determining the correlation values. Initially, bivariate analysis and later, multivariate analysis (negative binomial regression) after adjusting for confounders were performed to comment on the relation between the oral hygiene and oral health status. All the data entry and analysis were done using SPSS (IBM, USA) version 24.

## 3. Results

The study sample comprised of 500 (N) 12- and 13-year-old school going children, in which male and female were of equal proportion (50%). There was no gender difference in the demographic data evaluated in the current study (Table 1). The overall mean plaque score was observed to be on a higher side. With regards to location, there was no statistical variation between the plaque score among school children residing in the urban (mean = 0.66; SD = 0.33) or the rural (mean = 0.67; SD = 0.39) areas. Whereas in terms of gender, the mean plaque score in male school children (mean = 0.69; SD = 0.50) was slightly higher than the female school children (mean = 0.66; SD = 0.46). Also, the plaque score in the 13-year-olds (mean = 0.69; SD = 0.44) was slightly higher than the 12-year-olds (mean = 0.60; SD = 0.36) (Table 2).

Bivariate analysis revealed that oral hygiene habits, such as use of miswak (stick), flossing, and brushing, had strong negative relation with plaque formation. It is also observed that the children who brushed once daily (mean = 0.64; ß=−0.10; CI = −0.16, −0.04) had higher plaque formation than the children who brushed twice daily (mean = 0.62; ß=−0.11; CI = −0.18, −0.04) (Table 3). On analyzing mean DMFT against dietary habits, a strong correlation between frequency of eating, number of snack between meals, and sugar consumption per day was observed. It suggested that increased frequency of snacks (*P*=0.05; ß=0.08; CI = −0.00, 0.09) and sugar consumption (*P*=0.01; ß=0.16; CI = 0.04, 0.27) among children per day significantly showed higher values of DMFT (Table 4).

Multivariate analysis was performed to check the effect of various independent variables on plaque and DMFT scores. It revealed that 13-year-old children had higher odds of plaque formation in comparison to 12-year-olds (*P*=0.05; OR = 0.06; CI = −0.00, 0.12). Also, plaque formation was more in the school children who were irregular in brushing their teeth in comparison to one-time brushers (*P*=0.00; OR = −0.06; CI = −0.14, 0.00) and two-time brushers (*P*=0.00; OR = −0.09; CI = −0.17, −0.00) per day (Table 5). Similarly, the odds of dental caries among the school children who were irregular in brushing their teeth were higher in contrast to the children brushing once (*P*=0.03; OR = 0.89; CI = 0.70, 1.12) or twice (*P*=0.03; OR = 0.80; CI = 0.64, 0.93) per day (Table 5).

## 4. Discussion

This cross-sectional study is first to determine the association between oral hygiene practices and oral health status among 12- and 13-year-old school children in Saudi Arabia. Researchers elsewhere have considered oral hygiene as a key factor associated with good oral health [4, 5, 19, 20], and in the current study, it is evident that both frequency of brushing and sugar consumption per day are significantly related to the oral health status of school children in Saudi Arabia.

Fewer gender differences were subject to identification in regards to various demographic items. Moreover, the study indicates that placing both genders at the identical educational level puts them at the same threshold of the oral health issues. Also, there is no considerable difference between boys and girls when it comes to the elements of oral health status. The overall DMFT score among the school children aged 12 and 13 years in Jazan region of Saudi Arabia was observed to be considerably high. This finding is in accordance with a recent systematic review which reported that the mean DMFT of 70% permanent dentition among school going children in Saudi Arabia is 3.5 [21]. In contrast to this, a school-based survey conducted in the neighboring Arab country revealed a mean DMFT of 0.42 among 12-year-olds [22].

The current study also portrayed that a large proportion of children (70%) were from urban areas while a small proportion (30%) were from rural settings. Authors presumed that the use of miswak or chewing stick would be a common practice among the Arab children and would be the only practice by those residing in the rural regions. However, this was not the case with the findings from the current study. Arguably, it could be stated that brushing is common for those children coming from urbanized centers, but this may not be true always as one study conducted among children residing in rural region of southern Saudi Arabia showed that children residing in both the rural as well as urban regions used tooth-brush along with miswak to clean their teeth [23]. It is also seen that a good percentage of children in the current study reported using toothbrush for cleaning their teeth in comparison to miswak, and this shows that there is a general awareness on oral hygiene among children in Jazan region of Saudi Arabia, but another study conducted in Al-Jouf region of Saudi Arabia reported otherwise [24]. However, to confirm the findings, there is a need to ensure that these self-administered reports are confirmed through various objective assessments.

Core findings of this study revealed that there is a clear association between frequency of tooth brushing and sugar consumption with both: accumulation of plaque and dental caries. One part of this finding, that is, frequency of sugar consumption and its association with dental caries is supported by another study performed in Jazan region [17]. But, there are no previous studies conducted in or around this region among high school children in order to compare the oral hygiene practices with dental caries. However, studies conducted in other developing nations in the Asian continent displayed similar findings [25, 26]. We hope that the current study will aid in initiating future publications to address the oral hygiene issues surrounding oral health status among children in this region.

Strengths of the study are the selection of study participants was random and an appropriate sampling strategy was ensured to obtain a near representative sample, the questionnaire used was prevalidated and in addition, and clinical examination was performed to report oral health status. Also, the study included diverse group of children who attended the annual dental camp. Thus, unlike most previous studies in the kingdom that recruited participants from a specified location, our results can be more generalized to young Saudi population in Jazan. A limitation of this study is its cross-sectional design, and it is recommended that more studies with robust longitudinal design should be performed in future to provide stronger evidences.

To conclude, dental caries status of high school children in Jazan region of Saudi Arabia needs to be improved. The dental public health practitioners here should consider the effect of oral hygiene practices on oral health status in order to design the future health promotion interventions.

## Figures and Tables

**Table 1:** Gender variation in the demographic characteristics.

Variable^∗^	Gender		Total
	Male	Female	
Age			
12-year-old	137 (49%)	138 (51%)	275 (55%)
13-year-old	112 (49%)	113 (51%)	225 (45%)

Location			
Urban	215 (43.0%)	233 (46.5%)	448 (89.5%)
Rural	15 (3.0%)	38 (7.5%)	53 (10.5%)

Father's education			
No formal schooling	3 (0.5%)	3 (0.5%)	5 (1%)
Primary school	15 (3.0%)	3 (0.5%)	18 (3.5%)
Secondary school	8 (1.5%)	13 (2.5%)	20 (4%)
High school	63 (12.5%)	78 (15.5%)	140 (28%)
University	143 (28.5%)	175 (35.0%)	318 (63.5%)

Mother's education			
No formal schooling	3 (0.5%)	3 (0.5%)	5 (1%)
Primary school	13 (2.5%)	18 (3.5%)	30 (6%)
Secondary school	3 (0.5%)	13 (2.5%)	15 (3%)
High school	40 (8.0%)	60 (12.0%)	100 (20%)
University	173 (34.5%)	178 (35.5%)	350 (70%)

Mother occupation			
Full time	75 (15.0%)	93 (18.5%)	168 (33.5%)
Part time	25 (5.0%)	13 (2.5%)	38 (7.5%)
House maker	130 (26.0%)	165 (33.0%)	295 (59%)

^∗^Gender differentiation was not statistically significant.

**Table 2:** Mean plaque score (OHI-S), by age, gender, and location.

Variable	*N* (%)	Plaque mean (SD)
Age		
12-year-old	275 (55)	0.60 (0.36)
13-year-old	225 (45)	0.69 (0.44)

Gender^$^		
Male	250 (50)	0.69 (0.50)
Female	250 (50)	0.66 (0.46)

Location^$^		
Urban	338 (67.5)	0.66 (0.33)
Rural	163 (32.5)	0.67 (0.39)

^$^No significant difference in the mean plaque score.

**Table 3:** Mean plaque score and differences in scores by oral hygiene habits.

Oral hygiene habits	*N* (%)	Mean plaque score (SD)	Difference (95% CI)	*P*
Miswak	156 (31.2)	0.87 (0.5)	−0.09 (−0.17, −0.01)	0.04^∗^
Flossing	51 (10.1)	0.64 (0.4)	−0.02 (−0.10, 0.05)	0.06
Brushing				
Irregular	135 (27)	0.75 (0.6)	0.00	0.02^∗^
Once	158 (31.5)	0.64 (0.6)	−0.10 (−0.16, −0.04)	
Twice or more	208 (41.5)	0.62 (0.5)	−0.11 (−0.18, −0.04)	

^∗^Significant relation between the mean plaque score and oral hygiene habit.

**Table 4:** Mean DMFT score and differences in scores by dietary habits.

Dietary habits	*N* (%)	Mean DMFT (SD)	Difference (95% CI)	*P*
Frequency of eating/day				
1-2 times	77 (15.3)	0.73 (0.9)	0.00	0.05^∗^
3-4 times	326 (65.2)	0.63 (0.4)	−0.09 (−0.18, −0.02)	
More than 4 times	98 (19.5)	0.71 (0.8)	−0.02 (−0.09, 0.06)	

Snack between meals/day				
Once	189 (37.8)	0.62 (0.5)	0.00	0.05^∗^
Twice	200 (40.0)	0.70 (0.5)	0.06 (0.01, 0.11)	
Three or more	111 (22.2)	0.70 (0.7)	0.08 (−0.00, 0.09)	

Sugar consumption level/day				
Low	169 (33.7)	0.58 (0.5)	0.00	0.01^∗^
Moderate	206 (41.2)	0.66 (0.5)	0.07 (0.00, 0.15)	
High	126 (25.1)	0.74 (0.6)	0.16 (0.04, 0.27)	

CI: confidence interval. SD: standard deviation. ^∗^Significant relation between the mean DMFT score and dietary habits.

**Table 5:** Multivariate model for the association between decay, missing, filled, and plaque.

Variables	*N* (%)	Plaque adjusted OR (95% CI)	*P*	DMFT adjusted OR (95% CI)	*P*
Age					
12-year-old	275 (55)	0.00	0.05^∗^	1.00	0.18
13-year-old	225 (45)	0.06 (−0.00, 0.12)		1.12 (0.93, 1.35)	

Gender					
Male	250 (50)	0.00	0.28	1.00	
Female	250 (50)	−0.04 (−0.11, 0.12)		0.92 (0.81, 1.06)	

Mothers education					
University	128 (25.6)	0.00	0.77	1.00	0.26
High school	240 (47.9)	−0.02 (−0.11, 0.06)		0.97 (0.80, 1.17)	
Primary school	133 (26.5)	0.01 (−0.10, 0.13)		1.11 (0.92, 1.33)	

Snacks between meals/day					
Once	189 (37.8)	0.00	0.30	1.00	0.01^∗^
Twice	202 (40.4)	0.03 (−0.02, 0.09)		1.06 (0.86, 1.31)	
Three or more	109 (21.8)	0.06 (−0.03, 0.15)		1.25 (1.00, 1.57)	

Sugar consumption level/day					
Low	168 (33.6)	0.00	0.03^∗^	1.00	0.00^∗^
Moderate	207 (41.4)	0.07 (0.00, 0.14)		1.06 (0.86, 1.31)	
High	125 (25.0)	0.13 (0.02, 0.24)		1.25 (1.00, 1.57)	

Frequency of brushing/day					
Irregular	136 (27.1)	0.00	0.00^∗^	1.00	0.03^∗^
Once	159 (31.7)	−0.06 (−0.14, 0.00)		0.89 (0.70, 1.12)	
Twice or more	206 (41.2)	−0.09 (−0.17, −0.00)		0.80 (0.64, 0.93)	

^∗^Significant relation of the assessed independent variables with plaque and DMFT scores.
